# Simultaneous integrated boost with helical arc radiotherapy of total skin (HEARTS) to treat cutaneous manifestations of advanced, therapy-refractory cutaneous lymphoma and leukemia - dosimetry comparison of different regimens and clinical application

**DOI:** 10.1186/s13014-019-1220-5

**Published:** 2019-01-28

**Authors:** Chen-Hsi Hsieh, Hui-Ju Tien, Yuan-Bin Yu, Yuan-Hung Wu, Pei-Wei Shueng, Yueh-Feng Lu, Shan-Ying Wang, Li-Ying Wang

**Affiliations:** 1 Division of Radiation Oncology, Department of Radiology, Far Eastern Memorial Hospital, New Taipei City, Taiwan; 2 Faculty of Medicine, School of Medicine, National Yang-Ming University, Taipei, Taiwan; 3 Institute of Traditional Medicine, School of Medicine, National Yang-Ming University, Taipei, Taiwan; 4 Department of Biomedical Imaging and Radiological Science, National Yang- Ming University, Taipei, Taiwan; 5 Division of Oncology & Hematology, Far Eastern Memorial Hospital, New Taipei City, Taiwan; 6 Division of Radiation Oncology, Department of Oncology, Taipei Veterans General Hospital, Taipei, Taiwan; 7 Institute of Public Health, National Yang-Ming University, Taipei, Taiwan; 8 Division of Nuclear Medicine, Department of Radiology, Far Eastern Memorial Hospital, New Taipei City, Taiwan; 9 School and Graduate Institute of Physical Therapy, College of Medicine, National Taiwan University, Taipei, Taiwan; 10 Physical Therapy Center, National Taiwan University Hospital, Taipei, Taiwan

**Keywords:** HEARTS, Helical tomotherapy, Lymphoma, Leukemia, Total skin electron beam therapy

## Abstract

**Background:**

Helical irradiation of the total skin (HITS) was modified as simultaneous integrated boost (SIB)-helical arc radiotherapy of total skin (HEARTS) technique and applied to an acute myeloid leukemia (AML) patient with disseminated leukemia cutis.

**Methods:**

The original HITS plan was revised for different regimens, i.e. HEARTS, low-dose HEARTS and SIB-HEARTS. The uniformity index (UI), conformity index (CI), and dose of organs at risk (OARs) were used to evaluate the plans. Additionally, the SIB-HEART (21/15 Gy) was delivered to the total skin and chloromas.

**Results:**

No significant differences were observed for the CI and UI between HITS and HEARTS regimens. Compared with HITS, the reduced mean doses to various bone marrows ranged from 17 to 88%. The mean OARs doses for the head, chest and abdomen of a patient with AML treated with SIB-HEARTS (21/15 Gy) were 2.1 to 21.9 Gy, 1.8 to 7.8 Gy and 1.7 to 3.3 Gy, respectively. No severe adverse effects were noted except for grade 4 leukocytopenia and thrombocytopenia.

**Conclusion:**

HEARTS and different regimens reduced the dose to OARs and bone marrow while maintaining the uniformity and conformity. SIB-HEARTS deliveries different doses to the total skin and enlarged tumors simultaneously.

**Trial registration:**

Retrospectively registered and approved by the Institutional Review Board of our hospital (FEMH-106151-C).

## Introduction

Total skin electron beam therapy (TSEBT) is used to deliver 2 Gy (Gy) of radiation by electron beam energies of 3–4 MeV over a two-day period and treatment is 4 days per week up to a total dose of 30 to 36 Gy over an 8- to 10-week period [1]. However, dose deviation of 32 to 124% occurs in specific areas of the body during TSEBT, which contributes to treatment failure and poor clinical responses in patients with advanced disease [2–4].

**Fig. 1:**
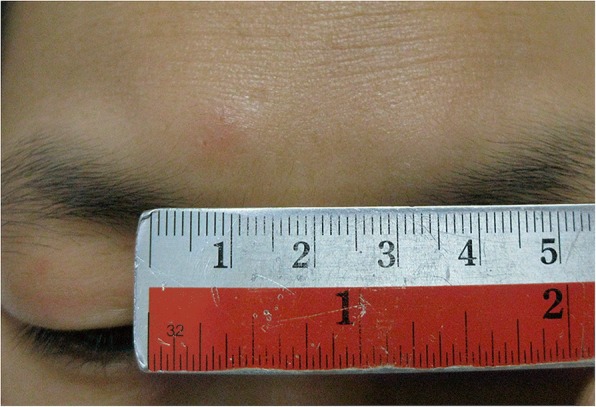
New growth patches in the right eyebrow and upper eyelid were noted in a patient treated by HITS after one year. The recurrent area was initially spared during irradiation

Recently, using helical tomotherapy (HT) to perform the helical irradiation of the total skin (HITS) technique was validated in a anthropomorphic phantom study [5] and was deemed acceptable for therapy-refractory cutaneous CD4+ T-cell lymphoma patient [6]. McGill University Health Center also used HT to treat the total skin of an infant diagnosed with refractory acute myeloid leukemia (AML) with extensive cutaneous involvement [7]. However, in a case previously reported by our team who suffered the new growth patches on the right eyebrow and upper eyelid one year after HITS treatment where were spared initially (Fig. 1), although other skins area remained intake and free from recurrence after one year and 8 months. This patient also exhibited grade 4 leukocytopenia and thrombocytopenia after treatment.

The outcome described above indicates complete skin irradiation is preferred over avoiding facial irradiation, which decreases adverse effects but increases failure risk. Recently, attention has been focused on lower-dose (10–12 Gy) TSEBT, which enables a shorter treatment duration with fewer side effects and the opportunity for retreatment if required [8–11]. Recently, the simultaneous-integrated boost (SIB) technique uses different dose levels to treat different targets into a single treatment session, rather than using sequential treatment plans [12]. The data support the possibility of achieving lower-dose total skin irradiation treated by HT combined with SIB technique to deliver a higher dose to the protruding tumor and a lower dose to the whole skin.

Leukemia cutis (also called chloromas) has been described in patients with acute myeloid leukemia (AML) and chronic myeloproliferative diseases [13]. The incidence of leukemia cutis in patients with AML is approximately 8% [14]. Studies confirm that radiotherapy can be applied to chloromas at doses of 10 to 26 Gy without significant adverse events [15–18]. Therefore, in this work, we revised the HITS plan using data regarding failure patterns and adverse effects to create an optimal treatment plan, called helical arc radiotherapy of total skin (HEARTS), to avoid possible failure and decrease toxicity. Low-dose HEARTS treatments with or without SIB were designed simultaneously to increase the tumor dose and decrease side effects. Moreover, a patient with AML refractory to allo-peripheral blood stem cell transplantation (PBSCT) presenting leukemia cutis treated with SIB-HEARTS was recruited to explore the availability of this technique in clinical applications.

## Materials and methods

### Phase I: Revision of the HITS plan comparing different regimens and dosimetry according to relapse patterns and adverse effects

#### HITS plan

The details of the HITS technique have been described previously. [6] In brief, the Philips Pinnacle^3^ treatment planning system was used for contouring. The clinical target volume (CTV) for a cutaneous lymphoma patient is the total skin with separated compartments except for the skin on the face. The CTV is defined as subcutaneous 0.5 cm from the skin. The inner margin of the planning target volume (PTV) is 0.5 cm. The outer margin of the PTV is three-dimensional (3D) expansion of 0.5 cm except for the arms, shoulders, and feet with 3D expansion of a 0.8 cm margin and the anterior side of the torso with 2D expansion of a 1.0 cm margin. The CTVs of bulging tumors were contoured independently to confirm adequate tumor. The prescription was 30 Gy in 40 fractions.

The central core complete block (CCCB) was contoured from only the head to the abdomen and maintained a 2.5 cm distance away from the PTV. The CCCB and the donut-shaped space between the PTV and CCCB (PC space) were also used to reduce internal organ dose and increase dose grading from the PTV. The hypothetical bolus was 1.0 to 1.5 cm which located from the skin in the body for a 0.5 cm margin covering the PTV to avoid over hitting during inverse optimization. The CT images and regions of interests were transferred to the Tomotherapy *Hi Art* Planning system (v. 4.2.3 Tomotherapy, Inc., Madison, Wisconsin, USA).

### Revision of HITS to HEARTS and low-dose HEARTS with or without the SIB technique

#### (A) HEARTS plan

The same CT images and contouring that were applied to the previous cutaneous lymphoma patient treated by HITS as reported [6] were used as basic HEARTS data. However, the CTV was revised by adding the contouring of the entire face to overcome previous local failure experiences (Fig. 1) and to ensure total skin coverage. The CTV_total skin_, CTV_face_ and CTV_eyelids_ were contoured from the skin using subcutaneous as 0.5, 0.4 and 0.3 cm, respectively. The inner margin of the PTV in total skin was reduced from 0.5 cm to 0.3 cm according to previous daily image-guided experience. The inner margin of the PTV in the eyelids was 0.1 cm to decrease possible damage to the eyes. The outer side margin for the PTV was maintained at 0.5 cm [6]. Similarly, the anterior PTV margin of the chest and abdomen adhered to the 0.8 cm rule with 3D expansion, and the thickness of the hypothetical bolus remained the same according to the rules mentioned above.

**Fig. 2:**
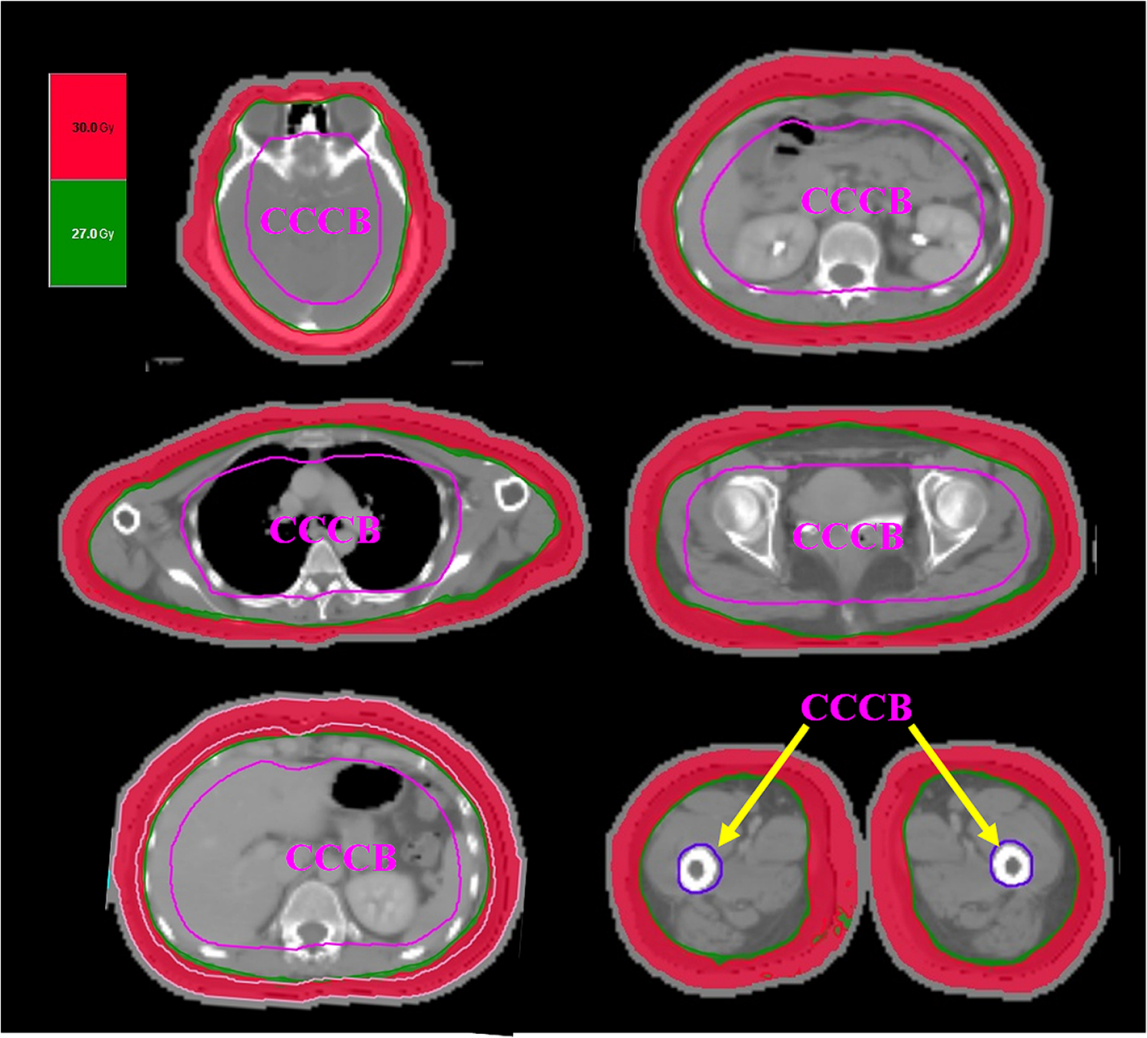
Revised helical irradiation of the total skin (HITS-30 Gy) to helical arc radiotherapy of total skin (HEARTS-30 Gy) by modifying the central core complete block (CCCB) in the head, chest, abdomen, pelvis, and thighs

Additionally, the CCCB was extended from the head to the thigh to reduce the integral dose (Fig. 2). The PC space was reduced from 2.5 cm to 2.2 cm due to the reduction of inner margin of the PTV and distance between CCCB and PTV. The CCCB in the pelvis and thigh was contoured in the femur bone. Bony structures such as the iliac bone, the cervical, thoracic, and lumbar spine; the femoral head and the pelvic bone minus the PTV were contoured separately from the references to reduce the bone marrow (BM) dose during optimization.

Thirty Gy with 40 fractions with 95% of the PTV was initially prescribed for HEARTS as HITS. The reference bony structures were assigned higher priority than the PC space to decrease the BM dose. The dose volume histograms (DVHs) were calculated for the target and individual organs at risk (OARs). The field width, pitch, and modulation factor used for HITS were 2.5 cm, 0.287, and 3.0, respectively.

#### (B) Low-dose HEARTS

Recently, low-dose regimens (10–12 Gy) have been applied to patients and demonstrated an effective reduction of disease burden in patients with mycosis fungoides [8, 9, 19, 20]. Herein, we modified HEARTS to examine a low-dose HEARTS regimen using 12 Gy for comparison.

#### (C) SIB-HEARTS

The SIB technique allows the planning and irradiation of different targets using different dose levels in a single treatment session instead of sequential treatment plans [12]. A patient with advanced cutaneous lymphoma and a bulging tumor might be treated using the SIB-HEARTS technique by applying 25 Gy to the protruding tumor and 12 Gy to total skin, which reduces toxicity during the entire treatment course while the protruding tumor is simultaneously treated at a higher dose.

### Plan evaluation

#### Conformity index (CI) and uniformity index (UI)

To compare HITS and different HEARTS regiments, the CI and UI were applied. The Paddick Conformity Index (PCI) was originally proposed by Paddick [21] to evaluate the tightness of fit of the PTV to the prescription isodose volume in treatment plans and was calculated as follows:

PCI = (TV_PIV_^2^) / (TV × PIV).

In which TV is the PTV volume, PIV is the treated volume enclosed by the prescription isodose surface, and TV_PIV_ is the volume of the PTV within the prescribed isodose. A PCI value close to unity means more conformity of the dose distribution to the target volume.

The UI is defined as *D*5%/*D*95%, in which *D*5% and *D*95% are the minimum doses delivered to 5 and 95% of the PTV, respectively, as previously reported [22].

#### Dose sparing for OARs

The mean dose was reduced to the lowest possible for all critical organs. The cervical spine, thoracic spine, lumbar spine, iliac bone, pelvic bone, and femur were contoured and expected to receive a mean dose less than 4.0 Gy to avoid BM toxicity [23].

### Phase II clinical application of SIB-HEARTS for advanced leukemia cutis

#### Patient characteristics

**Fig. 3:**
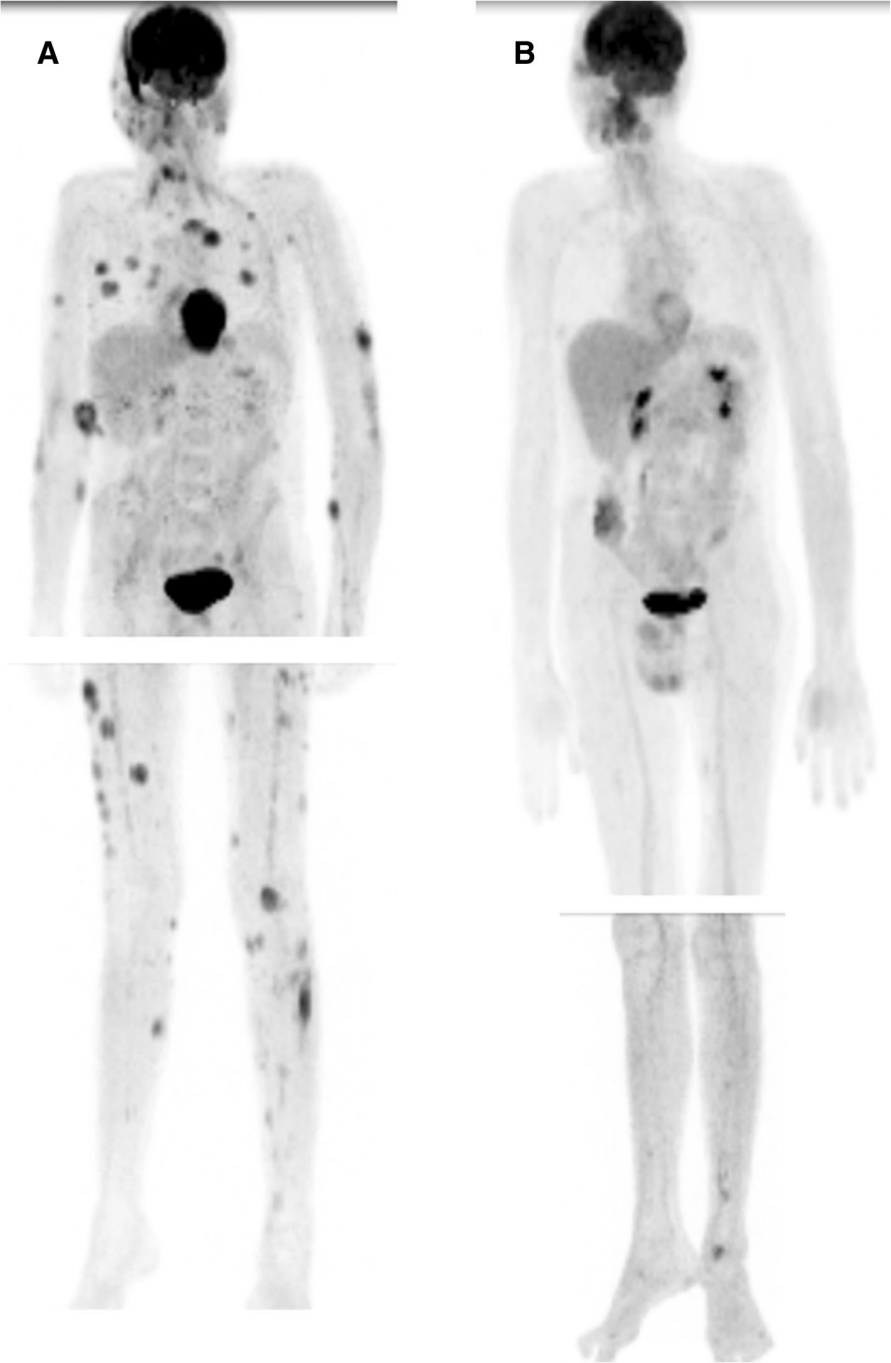
Disseminated leukemia cutis in patient with therapy-refractory acute myeloid leukemia (AML). **a** Positron emission tomography study (PET/CT) prior to SIB-HEARTS; the data showed 72 lesions throughout the body, 43 lesions in the bilateral legs and right vocal cord and bilateral testis involvement. The black spots indicate lesions on the skin. **b** PET/CT after SIB- HEARTS; the tumor regressed progressively in the entire body without further noduloplaques

In June, 2017, a 30-year-old male who was 186 cm tall visited our outpatient department due to progressive leukemia cutis for several months. The patient was diagnosed as AML with M1, and a normal karyotype without mutations of NPM-1 and FLT3-ITD. Between March 12 and 18 in 2016, the patient had been treated with cytarabine 100 mg/m^2^ plus idarubicin 12 mg/m^2^, high-dose cytarabine, etoposide plus busulfan and cyclophosphamide followed by allo-PBSCT. However, the patient suffered grade 3 acute graft-versus-host disease with extramedullary chloroma. Palliative radiotherapy with 20 Gy in 10 fractions was delivered to right nose and bilateral lower eyelids with nearly complete regression. Unfortunately, the patient underwent the progressed disease condition and disseminated chloroma on the body, the patient was referred to our hospital for total skin irradiation. When the patient visited our hospital, positron emission tomography-computed tomography (PET/CT) was performed and showed 72 lesions throughout the body, 43 lesions over the bilateral legs and right vocal cord and bilateral testis involvement (Fig. 3a).

#### CT simulation

A whole body diving suit was customized and included the head and extremities. The gloves, socks and head hood were also designed for the extremities and head. The type-S™ head and neck immobilization system (CIVCO radiotherapy, Orange City, Iowa, USA) was connected using 170 cm Body Vac Cushion (Klarity Medical, Newark, OH, USA) to immobilize the patient’s whole body position. The isocenters of the plans for the upper torso and the extremities were located in the chest and 10 cm above the knee, respectively. Two setup centers were located in the central areas of the eyebrows (head) and umbilicus (pelvis). The junction line of the plans for the upper torso and lower extremities were located 5 cm above the setup center of the lower extremities. Two CT image sets including the torso and lower extremities were acquired from the top of the head to the middle thigh and from the toe to the middle thigh, respectively. The slice thicknesses were 0.5 cm for the torso and lower extremity CT scans.

#### Design for the SIB-HEARTS technique

All of the lesions were contoured after fusion of the PET/CT images with the CT simulation images. Before applying the SIB-HEARTS technique, the previous HITS plan was redesigned in terms of HEARTS, low-dose HEARTS and SIB- HEARTS to confirm decreasing doses to the OARs with the similar uniformity and conformity. The SIB-HEARTS technique was subsequently used to treat the total skin with simultaneous lesion boost. The prescription for leukemia cutis in SIB-HEARTS (21/15 Gy) was 21 Gy to the lesions and 15 Gy to the total skin in 15 fractions and the minimal requirement of prescription dose for TV should be achieved. The plan was performed by Tomotherapy *Hi Art* Planning system (v. 5.1.3 Tomotherapy, Inc., Madison, Wisconsin, USA). The field width, pitch, and modulation factor (MF) used in SIB-HEARTS were 2.5 cm, 0.215, and 2.8, respectively.

#### Surface dose measurement

**Fig. 4:**
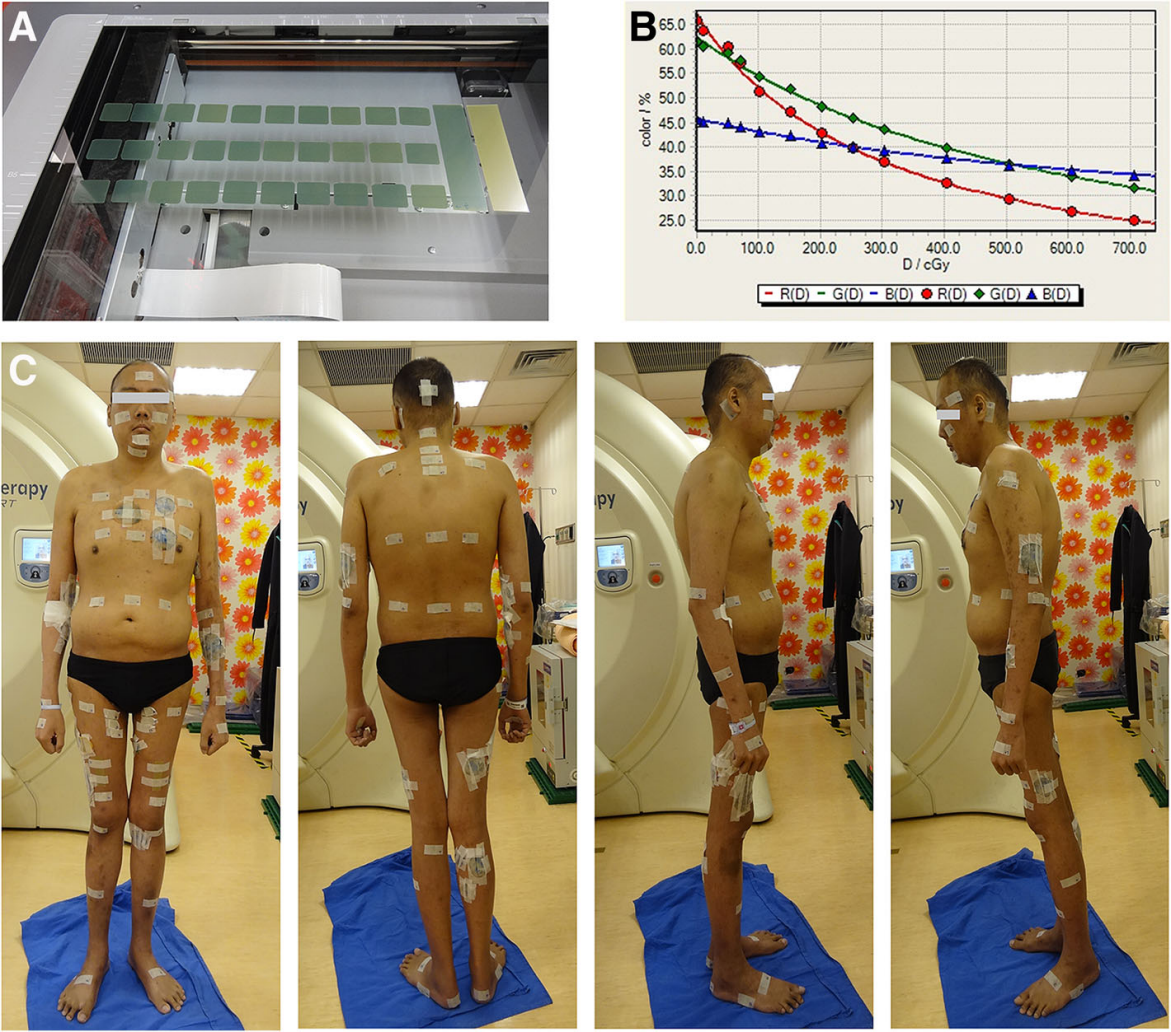
The Gafchromic EBT3 films with 2.5 × 3.0 cm^2^ sheets were used to measure the surface dose of body. **a** Single-scan protocol with measured films (2.5 × 3.0 cm^2^), background film (3 × 10 cm^2^) and absolute dose film (3 × 10 cm^2^). **b** Dose-response curves of Gafchromic EBT3 film in red, blue, and green color channel. **c** The distribution of EBT3 films on patient’s body

The Gafchromic EBT3 films with 2.5 × 3.0 cm^2^ sheets were used to measure the surface dose of body. The EBT3 films were digitized with a single scan by Epson 11000XL scanner and analyzed by Film QA Pro software (Film QA Pro 2015, version 5.0, Ashland Inc., Wayne, NJ, USA) [24]. The scanning and analytic process, dose-response calibration curves, and the distribution of EBT3 films in patient’s body were shown in Fig. 4. There were several lesions in the left anterior shoulder, chest and forearm near lateral abdomen. To confirm the dose deviation may persist in the area near lesions in the SIB technique, the locations for EBT3 films were designed as far away the lesions and close to the lesions in the first and second measurement courses, respectively.

## Results

### Dose comparison for HITS, HEARTS, low-dose HEARTS and *SIB-HEARTS*

**Fig. 5:**
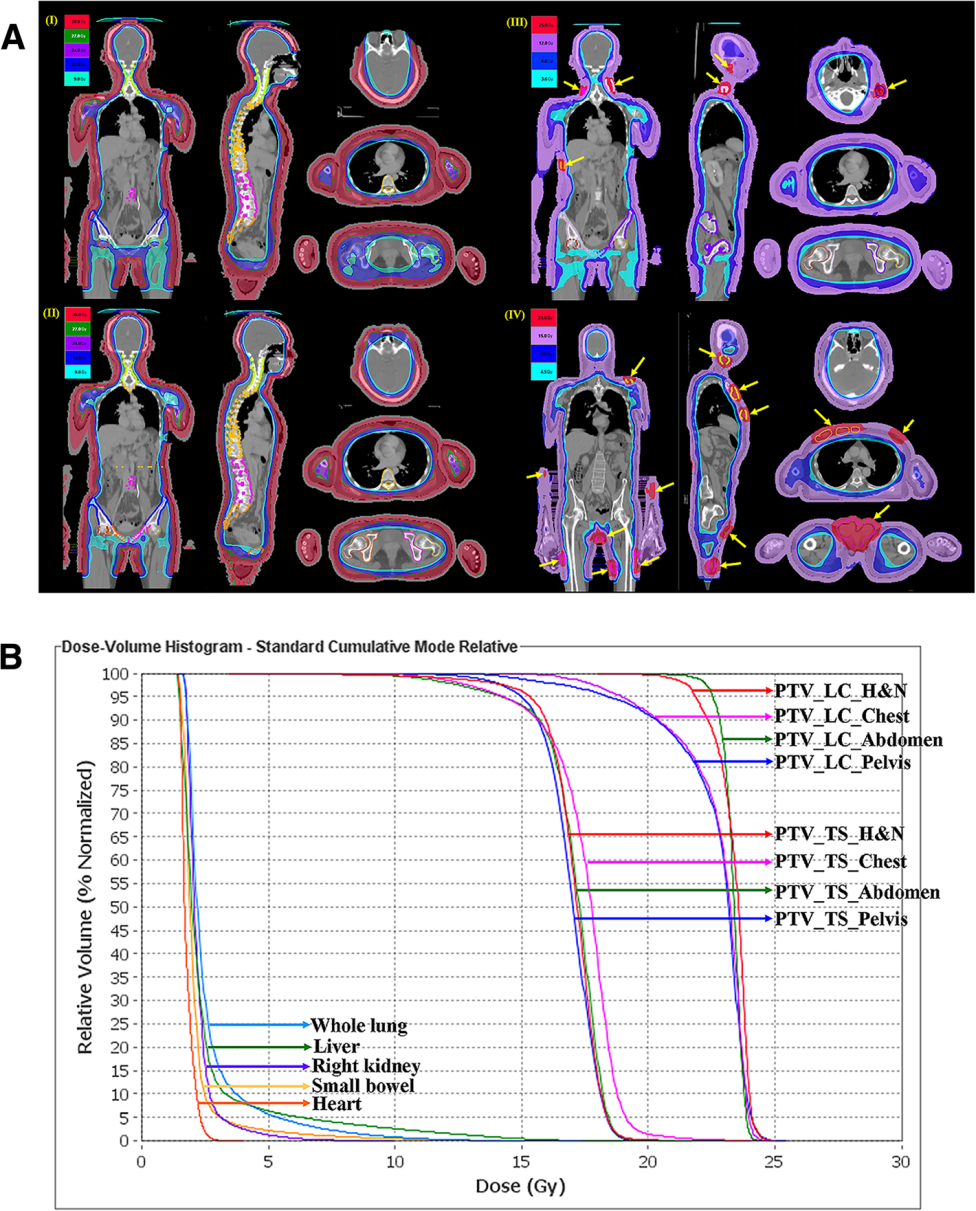
Revised helical irradiation of the total skin (HITS-30 Gy) to helical arc radiotherapy of total skin (HEARTS-30 Gy) and low-dose HEARTS (12 Gy) with or without the simultaneous integrated boost (SIB-25/12 Gy) technique according to the failure patterns and adverse effects from a previous case treated by HITS. **a** Isodose distribution in different regimens of total skin irradiation, (I) HITS; (II) HEARTS; (III) SIB-HEARTS (25/12 Gy); (IV) clinical application for SIB-HEARTS (21/15 Gy). The yellow arrows indicate lesions treated with SIB technique. **b** Dose-volume histogram (DVH) in clinical SIB-HEARTS. (PTV: Planning target volume; LC: Leukemia cutis; TS: Total skin; H&N: Head and neck)

Technique comparisons for HITS and HEARTS are presented in Table 1. The isodose distributions in the axial, sagittal and coronal views obtained with different plans are shown in Fig. 5a. The PCI and UI for the skin of the head, chest, abdomen and pelvis with upper thigh in different plans are listed in Table 2. The PCI and UI were similar between these plans and in the clinical application. Multiple setup centers were present in the HITS and HEARTS plan rather than one center, as reported by Sarfehnia A et al. [7]. For an adult patient, multiple setup centers with an image-guided system ensures reproducibility between CT images and treatment accuracy for megavoltage- computed tomography and also overcomes the geometric miss to avoid marginal failure. Moreover, the maximum average value of registration for the upper torso versus lower extremities in different translation directions were 2.8 mm versus 0.9 mm for pretreatment and 0.7 mm versus 0.6 mm for posttreatment, respectively [6].

**Table 1:** Technique comparison of helical irradiation of total skin (HITS) and helical arc radiotherapy of total skin (HEARTS)

	HITS	HEARTS
Target delineation	Face sparing	Total skin without sparing
Location of CCCB	Head& chest& abdomen	Head& chest& abdomen & pelvis & bilateral upper one-third of femur
Distance between CCCB and PTV	2.5 cm	2.2 cm
Inner margin of PTV	0.5 cm	0.3 cm
Bone marrow targeting	Bony structures included in PC space	Independent bony structures adjacent with PC space
Priority of RARs	PC space	Independent bony structure > PC space

Abbreviation: *CCCB* Central core complete block, *PC* space Space between PTV and CCCB, *PTV* Planning target volume, *RARs* Regions at risk

**Table 2:** The comparison of the Paddick Conformity Index (PCI) and uniformity index (UI) in different portions of the torso with different techniques and in the clinical application of SIB-HEARTS

	Head		Chest		Abdomen		Pelvis&upper thigh	
	PCI	UI	PCI	UI	PCI	UI	PCI	UI
Planning comparison								
HITS	0.70	1.12	0.73	1.12	0.72	1.08	0.72	1.12
HEARTS	0.65	1.16	0.71	1.12	0.70	1.08	0.70	1.15
Low-dose HEARTS	0.66	1.12	0.72	1.12	0.69	1.11	0.70	1.13
SIB-HEARTS (25/12Gy)	0.63	1.38	0.70	1.25	0.66	1.25	0.68	1.31
Clinical application								
SIB-HEARTS (21/15 Gy)	0.67	1.20	0.59	1.35	0.61	1.31	0.69	1.23

Abbreviation: *HEARTS* Helical arc radiotherapy of the total skin, *HITS* Helical irradiation of the total skin, *SIB* Simultaneous integrated boost

### Dosage to OARs

The mean doses of HITS, HEARTS regiments and the clinical application of SIB-HEARTS to a fresh case with various OARs are listed in Tables 3. Compared with HITS, several of the doses for the head, chest and abdomen were reduced in HEARTS, low-dose HEARTS and SIB-HEARTS by 6 to 71%, 62 to 88%, and 53 to 87%, respectively. The mean doses to the different bones in HEARTS, low-dose HEARTS and SIB-HEARTS were reduced by 17–72%, 67–88% and 53–87%, respectively. The mean doses of SIB-HEARTS (21/15 Gy) that were applied to a patient with AML had OARs of the head, chest and abdomen that were 2.1 to 21.9 Gy, 1.8 to 7.8 Gy and 1.7 to 3.3 Gy, respectively (Table 3). The dose volume histogram (DVH) to the target and OARs for a patient with leukemia cutis treated by SIB-HEARTS (21/15 Gy) is shown in Fig. 5b and the TVs got more than 15 to 20% of nominal prescript doses due to the minimal requirement of prescription dose as 21/15 Gy for TV should be achieved.

**Table 3:** Calculated doses to organs at risk for HITS, HEARTS, low-dose HEARTS, and SIB-HEARTS and clinical application of SIB-HEARTS to leukemia cutis with various OARs for the head, chest and abdomen

Organs at risk	Original planning	Revised planning			AML with leukemia cutis
	HITS (30 Gy)	HEARTS (30 Gy)	Low-dose HEARTS (12 Gy)	SIB- HEARTS (25/12 Gy)	SIB- HEARTS (21/15 Gy)
	Mean dose or maximal dose (Gy)				
Whole brain	8.0	6.8	2.9	2.0	2.1
Brain stem (max.)	2.5	3.2	1.3	1.4	1.6
Spinal cord (max.)	3.8	3.8	1.5	1.8	3.5
Right lens (max.)	2.1	29.7	13.1	12.2	8.7
Left lens (max.)	2.2	30.6	12.9	12.7	7.8
Right eye	3.8	21.8	9.2	9.0	3.8
Left eye	4.1	21.6	9.3	9.4	4.1
Right parotid gland	29.3	10.4	9.3	6.8	9.0
Left parotid gland	29.9	20.2	8.8	12.2	12.2
Lips	15.8	33.1	13.1	12.4	17.0
Oral cavity	8.7	8.2	3.3	2.4	2.7
Pharynx	5.2	4.5	1.8	1.6	4.0
Larynx	23.2	17.2	7.4	6.1	21.9
Trachea	13.7	10.4	4.4	4.8	5.0
Thyroid	24.7	20.2	8.6	11.4	12.9
Esophagus-upper part	8.1	4.6	2.0	2.4	7.8
Esophagus-middle part	3.1	2.6	1.0	1.1	1.8
Esophagus-lower part	2.9	2.6	1.0	1.0	1.6
Right lung	4.7	3.4	1.5	1.6	2.5
Left lung	4.5	3.4	1.5	1.5	2.8
Whole lung	4.6	3.7	1.5	1.5	2.6
Heart	3.3	2.9	1.1	1.2	1.8
Liver	5.2	3.9	1.6	1.9	2.5
Spleen	6.8	5.2	2.2	2.0	3.3
Right kidney	3.9	3.3	1.3	1.5	2.2
Left kidney	4.3	3.5	1.4	1.4	2.5
Bladder	11.2	4.7	1.9	1.7	2.1
Rectum	8.5	5.5	2.2	2.1	2.5
Uterus&ovary	4.3	3.2	1.3	1.3	–
Cervix&vagina	15.7	9.3	3.8	3.7	–
Intestine	4.7	3.5	1.4	1.5	2.1
Stomach	3.6	3.0	1.2	1.3	1.7
Bone marrow in different parts					
Cervical spine	5.8	3.6	1.5	1.9	2.2
Thoracic spine	6.3	3.6	1.4	1.5	2.3
Lumbar spine	4.0	3.3	1.3	1.3	1.9
Sacrum	4.8	4.0	1.6	1.4	3.0
Right iliac crest	8.9	6.1	2.4	2.1	3.6
Left iliac crest	8.5	6.2	2.5	4.0	3.1
Right femur	12.3	3.5	1.5	1.6	2.2
Left femur	10.3	3.4	1.4	1.5	2.9
Right pelvic bone	13.1	5.7	2.3	2.2	3.0
Left pelvic bone	12.2	5.8	2.3	3.6	2.8

### Response and toxicities for a patient with leukemia cutis treated by SIB-HEARTS

Patient data were collected with the approval of the Institutional Review Board of our hospital (FEMH-106151-C). Fifteen Gy to the total skin with 21 Gy to the tumor in 15 fractions were delivered to the patient from June 13, 2017 to July 3, 2017. The tumor regressed progressively over the entire body. As demonstrated by pathology, the lesions over the left arm were dermal sclerosis without tumor cells. Grade 2 dermatitis and mucositis were noted during treatment. Grade I xerostomia, fatigue, anemia, thrombocytopenia, leukocytopenia and body weight loss (51 to 46 kg) were noted during the procedure. Complete recovery from hoarseness was observed after treatment. No onycholysis, epitheliolysis, phlyctenules, tumor lysis syndrome, fever, vomiting, dyspnea, edema of the extremities, or diarrhea occurred during treatment. No decreased abnormal renal function was noted during or after treatment. Anhidrosis was noted two weeks following completion of the treatment and lasted for 6 weeks. Transient alopecia was noted after SIB-HEARTS. Desquamation with skin itching following three weeks completion of the treatment was also noted.

**Fig. 6:**
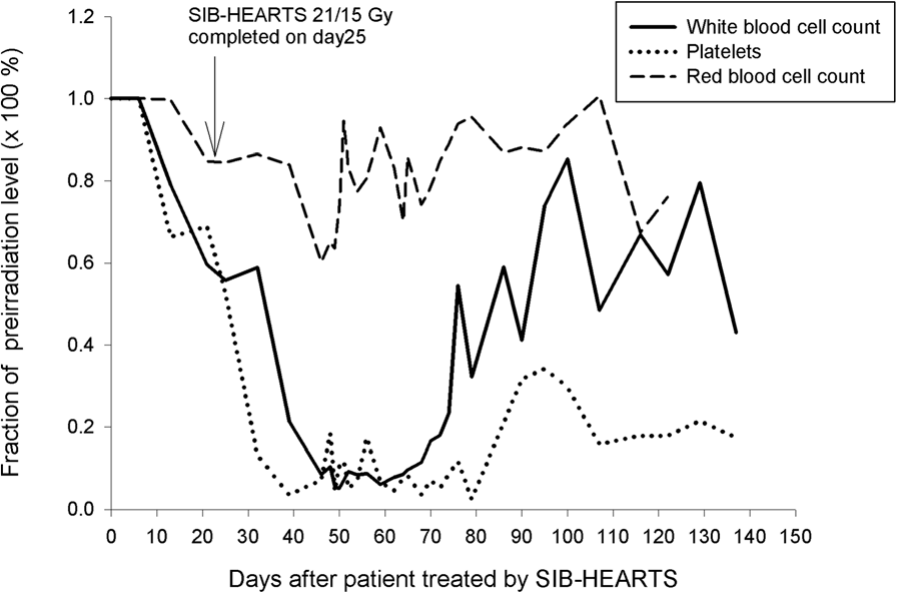
Time course of blood cell count during and after treatment

However, grade 4 leukocytopenia (510/μL) and thrombocytopenia (7 × 10^3^/μL) were noted post-treatment on day 17. The white blood cell (WBC) count dropped to a nadir on post-treatment day 21 (310/μL) and recovered to grade 1 (3250/μL) on post-treatment day 47. On post-treatment day 60, thrombocytopenia recovered to grade 2. (Fig. 6) Supportive measures were delivered, including partial parenteral nutrition, antibiotics, hematopoietic colony-stimulating factors and yeast-derived 1,3/1,6 glucopolysaccharide.

Day 28 after treatment, total bilirubin increased to 3.4 mg/dL and increased to 13.2 mg/dL 14 days later with poor appetite and nausea. Abdominal echo showed a dilated common bile duct and intrahepatic duct. Drug-related acute cholecystitis was suspected, thus antibiotics were supplied, and partial parenteral nutrition was titrated. However, total bilirubin was quite high (9.7 mg/dL) on post- treatment day 57. An abdominal MRI showed 2.4-cm segmental narrowing at the middle third of the common bile duct that was compressed by an enlarged lymph node in the porta hepatis and only minimal dilatation of the IHDs. Biopsy data showed immature myeloid cells involved by leukemia. The lesion of the hepatic hilum was treated with 30 Gy in 5 fractions. However, total bilirubin remained high, and ERBD (Boston Scientific 10 Fr and 7 cm) insertion was performed. After SIB-HEARTS, the following PET-CT report showed complete regression of previous numerous skin or subcutaneous soft tissues over the whole body, and no other definite sites of abnormal hypermetabolism were noted in the hepatic hilum, but new abnormal hypermetabolic lesions were observed at the right paraoropharyngeal wall and mid-ascending colon. (Fig. 3b).

The patient returned to the original hospital for additional chemotherapy. DNA was analyzed using PCR-base short tandem repeats analysis and dropped to 98%, confirming relapse. Therefore salvage chemotherapy was performed in the original hospital. However, the patient suffered sepsis with hepatic failure (total bilirubin, 54 mg/dL) and expired on November 6, 2017.

### Surface doses to the skin in a patient with leukemia cutis treated by SIB-HEARTS (21/15 Gy)

The surface doses to the skin are listed in Table 4. The surface dose in the lesions was 120–133% of prescribed dose (ranging from 167.4 to 185.7 cGy). The surface doses measured by exposed EBT3 films in the head, chest, back, abdomen, right upper extremity, left upper extremity, right lower extremity and left lower extremity were 130–132%, 143–154%, 125–129%, 131–142%, 118–149%, 129–146%, 98–117% and 93–122% of the prescribed dose, respectively.

**Table 4:** Surface doses to the skin from head to toes in a patient with leukemia cutis treated by SIB-HEARTS (21/15 Gy) as measured with radiochromic EBT3 film

Site	Surface dose (cGy)/ fraction			% of prescription dose
	1st measurement	2nd measurement	Average	
Head				
Vertex of head	127.2	132.0	129.6	130%
Occipital	129.6	130.5	130.1	130%
Frontal	137.1	126.6	131.9	132%
Chest				
Right, upper	135.7	150.9	143.3	143%
Right, lower	140.6	167.9	154.3	154%
Back				
Middle, upper	134.9	122.0	128.5	129%
Middle, lower	120.4	128.7	124.6	125%
Abdomen				
Middle, anterior	130.7	131.8	131.3	131%
Middle, lateral	127.6	155.9	141.8	142%
Upper extremities, right				
Shoulder	106.1	128.9	117.5	118%
Elbow	155.0	142.4	148.7	149%
Hand	117.6	124.6	121.1	121%
Palm	134.1	138.1	136.1	136%
Upper extremities, left				
Shoulder	111.2	147.5	129.4	129%
Elbow	145.7	145.8	145.8	146%
Hand	124.4	141.9	133.2	133%
Palm	128.5	141.6	135.1	135%
Right side of lower extremities				
Thigh, anterior	120.6	114.2	117.4	117%
Thigh, medial	115.8	113.5	114.7	115%
Lower leg, anterior	110.0	110.2	110.1	110%
Foot	98.1	97.7	97.9	98%
Left side of lower extremity				
Thigh, anterior	127.8	114.6	121.2	121%
Thigh, medial	116.7	113.5	115.1	115%
Lower leg, anterior	113.1	130.1	121.6	122%
Foot	95.8	89.7	92.8	93%
Scrotum	142.2	134.5	138.4	138%
Right inguinal area	134.5	127.8	131.2	132%
Lesions				
Right chest lesion	184.6	186.0	185.3	132%
Left chest lesion	191.6	179.7	185.7	133%
Right forearm lesion	165.4	169.4	167.4	120%
Right thigh lesion	172.8	179.8	176.3	126%

## Discussion

The inhomogeneity of TSEBT throughout the skin surface is approximately 15% [25]. The deviations from the prescription dose in specific areas of the body ranged from 32 to 124% using TSEBT [2] and 95.7 to 123.8% in HITS [6]. In the SIB-HEARTS’ technique, the real surface dose in the lesions and total skin ranged from 120 to 133% and from 93 to 154% of prescription dose, respectively. Additionally, the UI and the PCI in different portions of the torso were 1.21–1.35 and 0.59–0.69, respectively. (Table 2) Additionally, an infant treated by total skin irradiation using HT showed 10% overestimation of the absorbed dose near the surface from the HT treatment planning [7]. However, dressing in the diving suit (3 mm thick) and covering the lesions with Polyflex II tissue equivalent material (Sammons Preston, Warrenville, IL, USA) and conformal material (R.P.D., Albertville, MN, USA) as a bolus successfully increased superficial doses [6]. Therefore, HITS or SIB-HEARTS overcome the problem of TSEBT inhomogeneity while precisely improving conformality and depth penetration with impressive treatment outcome [6].

The variation of surface dose that deliver by electron beam or photon beam with increasing incident angle has been studied by several authors and similar results on the increased surface dose from oblique incidence have been reported [26–30]. The oblique factors for 60° incidence angle are 1.19–1.30 for 6 MeV [26, 28] and 1.5 for 6 MV [30]. However, the penetration of electron beam falls off rapidly after Dmax but the photon beam falls off smoothly. These characteristics make electron beams obliquely incident onto surface with bolus results in a decreased surface dose relative to an unbolused surface but photon beam does not. The CCCB used in SIB-HEARTS or HITS techniques to restrict the photon beams to be obliquely incidence with hypothetical bolus that increases the superficial dose and make the measured dose range from 93 to 154% of prescription dose in the SIB-HEARTS and 95.7 to 123.8% in HITS [6]. However, the benefits limited the dose fall-off grading in the SIB technique especially there are big gap between both dose levels (21/15 Gy) like the current study. The data showed in the first measurement is the films putting on the far away lesion sites and the second measurement is the films setting on the sites close to lesions although these locations in the same area of body (Table 4). The obvious deviations between both calculated doses were noted by 15, 27, 28 and 36% in the right upper-chest, right lower-chest, lateral middle-abdomen and left shoulder, respectively.

The interval from a patient treated by HITS to HITS-treated area failure was one year and 8 months and this patient remained alive approximately 2.5 years after HITS-treatment [6]. The acute and late toxicities were acceptable and did not cause permanent damage, suggesting that HITS at a standard dose is a potential treatment choice for a refractory cutaneous lymphoma patient. However, the patient treated by HITS suffered from out-fields failure. The area was located in the right eyebrow and upper eyelid (Fig. 1). Certain factors and/or cellular interactions operate in the lesion skin to induce epidermotropism of the malignant cells [31] and contribute to the epidermal T-cell population, resulting in an increase in the dermal T-cell volume during the course of the disease [32]. Therefore, development of the HEARTS technique to replace HITS is required to prevent epidermotropism in the normal skin that interacts with lesions. The UI and PCI for the HEARTS different techniques were similar to those of HITS, which explains the reproducibility and similar properties observed in the clinical application of these plans (Table 2).

In a retrospective TSEBT study, the overall response rate associated with ranges of 10 to less than 20 Gy or 20 to less than 30 Gy were comparable to doses of 30 Gy or more [33]. Kamstrup et al. [9] reported results for patients treated with 10 Gy TSEBT. The overall response rate was 95% with a complete cutaneous response or a very good partial response rate documented in 57% of the patients. For the 12 Gy TSEBT trial, the overall response rate was 88% (29/33), including 9 patients with complete response [8]. Compared with HITS, the rate of dose reduction to the OARs in low-dose HEARTS techniques was 62 to 88%, indicating that low-dose HEARTS can be safely administered multiple times to patients with recurrent disease due to its acceptable toxicity profile.

For chloromas, Elsayad K et al. [15] reported that a radiation dose ≤ 26 Gy confers a comparable complete response rate (83%) to that conferred by > 26 Gy regimens (95%, *p* = 0.26). Bask et al. [16] recommended irradiating chloromas to at least 20 Gy as an appropriate regimen. Hall et al. [17] reported that 25 patients were treated with RT at a median dose of 24 Gy; and 44% of patients experienced partial relief, and 48% experienced complete symptomatic improvement without significant acute toxicities. Bakst R and Yahalom J also reported that 15 patients received TSEBT with a median dose of 16 Gy (range 6–24 Gy) and that 15% of patients showed a complete response to RT [34]. In this study, the patient showed complete response for all skin lesions to SIB-HEARTS (21/15 Gy), suggesting that the concept of SIB integration with HEARTS is a workable and pragmatic strategy (Fig. 3a and b).

The patient treated by HITS suffered from grade 4 leukocytopenia and thrombocytopenia [6]. Therefore we revised HITS as HEARTS to decrease doses to the BMs and critical organs. Compared with the HITS technique, the mean doses to BM in various locations were reduced in HEARTS, low-dose HEARTS and SIB-HEARTS by 17–72%, 67–88% and 53–87%, respectively. Theoretically, the hematologic toxicity could be reduced using the HEARTS regimens. No hematologic toxicity was noted for SIB-HEARTS (21/15 Gy) during the treatment days. However, the patient exhibited grade 4 leukocytopenia and thrombocytopenia after treatment was complete, although the received BM dose ranged from 1.9 to 3.6 Gy and was reduced by 38–82% when compared with the HITS plan dose (Table 3). Recently, Schaff et al. also reported two patients treated with HITS technique with bone marrow of a mean dose of < 2 Gy and experienced severe bone marrow suppression including grade 4 thrombocytopenia [35].

When humans and rodents receive total-body irradiation (TBI), death by hematopoietic syndrome in the first 30 days is primary due to neutropenia and thrombocytopenia [23, 36, 37]. For hematopoietic syndrome, depletion of hematopoietic progenitor cells of white blood cell and megakaryocyte lineages within 30 days lead to neutropenia and thrombocytopenia [36, 37]. Hematopoietic progenitor cells are more radiosensitive than pluripotent stem cells [38]. Mitotically active hematopoietic progenitors have a limited capacity to divide after a whole-body radiation dose greater than 2 to 3 Gy [39]. Although most BM progenitors are susceptible to cell death after sufficiently intense radiation doses, subpopulations of stem cells or accessory cells are selectively more radioresistant [40]. These radioresistant cells might play an important role in the recovery of hematopoiesis after exposure to doses as high as 6 Gy, albeit with a reduced capacity for self-renewal [23]. However, irradiated pluripotent stem cells required a long time (approximately 30 days) to be recruited and reconstitute neutrophils and platelets. [41] Therefore, the possible reasons for hematopoietic syndrome in patients treated by SIB-HEARTS or by HITS technique [6] are that the dose irradiated to the BM causes the hematopoietic progenitor cells depletion and slow pluripotent stem cells recruitment. Therefore, 30–40 days after completing SIB-HEART, pluripotent stem cells reconstitute the various BM lineages into the cell cycle, and the patient recovers progressively (Fig. 5). Prior to the recovery, prolonged therapy with cytokines, blood component transfusion, radioprotectors [42], and even stem-cell transplantation might be appropriate [23].

## Conclusion

The HEARTS regimens successfully reduced the doses to the OARs and BM and supplied the same consistent uniformity and conformity as the HITS technique. SIB-HEARTS simultaneously delivers different doses to total skin and enlarged tumors. SIB-HEARTS can overcome the inhomogeneity that was criticized in the TSEBT technique and provides a good reproducibility in the clinical practice which will provide guidance in the future to other clinical practitioners, i.e., the preparations of CT sim, the references for dose constrain and OARs and titration of dose for SIB-HEARTS patients. These findings should help doctors discuss risks and benefits with their patients for various courses of radiation therapy and inform shared decision-making between physicians and patients. Further studies are warranted to clarify the pros and cons of these new approaches in clinical applications.

## Abbreviations

3D: Three-dimensional
AML: Acute myeloid leukemia
BM: Bone marrow
CCCB: Central core complete block
CI: Conformity index
CTV: Clinical target volume
DVHs: Dose volume histograms
Gy: Gray
HEARTS: Helical arc radiotherapy of total skin
HITS: Helical irradiation of the total skin
HSCs: Pluripotent stem cells
HT: Helical tomotherapy
OARs: Organs at risk
PBSCT: Peripheral blood stem cell transplantation
PET-CT: Positron emission tomography-computed tomography
PTV: Planning target volume
SIB: Simultaneous integrated boost
TBI: Total-body irradiation
TSEBT: Total skin electron beam therapy
UI: Uniformity index
WBC: White blood cell
